# Brownian Motion and Thermophoresis Effects on MHD Three Dimensional Nanofluid Flow with Slip Conditions and Joule Dissipation Due to Porous Rotating Disk

**DOI:** 10.3390/molecules25030729

**Published:** 2020-02-07

**Authors:** Nasser Aedh Alreshidi, Zahir Shah, Abdullah Dawar, Poom Kumam, Meshal Shutaywi, Wiboonsak Watthayu

**Affiliations:** 1 College of Science Department of Mathematics Northern Border University, Arar 73222, Saudi Arabia; nasser.alreshidi@nbu.edu.sa; 2 Center of Excellence in Theoretical and Computational Science (TaCS-CoE), SCL 802 Fixed Point Laboratory, Science Laboratory Building, King Mongkut’s University of Technology Thonburi (KMUTT), 126 Pracha-Uthit Road, Bang Mod, Thrung Khru, Bangkok 10140, Thailand; zahir.sha@kmutt.ac.th; 3 Department of Mathematics, Abdul Wali Khan University Mardan 23200, Pakistan; abdullah.mathematician@gmail.com; 4 KMUTT Fixed Point Research Laboratory, Room SCL 802 Fixed Point Laboratory, Science Laboratory Building, Department of Mathematics, Faculty of Science, King Mongkut’s University of Technology Thonburi (KMUTT), Bangkok 10140, Thailand; wiboonsak.wat@kmutt.ac.th; 5 KMUTT-Fixed Point Theory and Applications Research Group, Theoretical and Computational Science Center (TaCS), Science Laboratory Building, Faculty of Science, King Mongkut’s University of Technology Thonburi (KMUTT), Bangkok 10140, Thailand; 6 Department of Medical Research, China Medical University Hospital, China Medical University, Taichung 40402, Taiwan; 7 Department of Mathematics College of Science & Arts Rabigh, King Abdul-Aziz University, Jeddah 21911, Saudi Arabia; mshutaywi@kau.edu.sa

**Keywords:** nanofluid, porous medium, MHD, viscous dissipation, slip effect, rotating disk, HAM, shooting

## Abstract

This paper examines the time independent and incompressible flow of magnetohydrodynamic (MHD) nanofluid through a porous rotating disc with velocity slip conditions. The mass and heat transmission with viscous dissipation is scrutinized. The proposed partial differential equations (PDEs) are converted to ordinary differential equation (ODEs) by mean of similarity variables. Analytical and numerical approaches are applied to examine the modeled problem and compared each other, which verify the validation of both approaches. The variation in the nanofluid flow due to physical parameters is revealed through graphs. It is witnessed that the fluid velocities decrease with the escalation in magnetic, velocity slip, and porosity parameters. The fluid temperature escalates with heightening in the Prandtl number, while other parameters have opposite impacts. The fluid concentration augments with the intensification in the thermophoresis parameter. The validity of the proposed model is presented through Tables.

## 1. Introduction 

Nanofluid is the suspension (mixture) of base fluid (water, gasoline oil, kerosene oil, ethylene glycol) and nanometer-sized particles, which is called nanofluid. Nanofluids are made of different martials, like metals (Ag, Au, Cu), carbon (CNTs, diamonds, graphite), metal nitrides and oxide ceramics (CuO, Al_2_O_3_), etc. In the current science era, nanofluid has turned in a significant constituency of research. Due to its extensive variety of applications in science, engineering, and technologies, like computers, heating and cooling devices, microelectronics, heat exchanger MHD micropumps, etc. Therefore, nanofluid flow in microchannel captivated the significant consideration of researchers. In the last several years, these fluids have been comprehensively use, one of them being ′nanofluid′. The word in this context nanofluid had first been actually invented by Choi [1], which characterizes the dilution of nanoscale materials in a base body fluid, like ethylene glycol, water, and oil. Makinde and Aziz [2] reported the heat transfer flow of nanofluid through the extending sheet. Turkyilmazoglu et al. [3] analyzed the cumulative consequences of the mass and heat transfer of nanofluids across a horizontal plate, together with radiation. Mustafa et al. [4] examined the boundary layer flow of nanofluid over a rashly stretched surface. Ashorynejad et al. [5] investigated the properties of MHD nanofluid flow and heat transmession. Murthy et al. [6] observed the thermal conduction transfer rate of stratified nanofluid coated with a non-dark porous medium thorough a horizontal layer. Rashidi et al. [7] demonstrated the entropy production of nanofluid in the existence of a magnetic field that is caused by a rotated porous disk. Tham et al. [8] examined the convection flow of gyrotactic microorganism-containing nanofluid to a solid sphere encoded in a porous medium. Aziz et al. [9] have reported convection heat transfer flow that is caused by nanofluid across a vertical flat plate comprising motile microorganisms. Shah et al. [10] numerically deliberated the heat transfer in MHD nanofluid with shape factor in permeable media. Zubair et al. [11] presented the MHD Casson nanofluid flow with entropy generation. Kumam et al. [12] scrutinized the radiative flow of MHD Casson nanofluid with entropy generation in rotating channels. Shah et al. [13] studied the ferrofluid with Cattaeo heat flux by means of thermal conductivity model.

Air cleaning machines, centrifugal filtration, food processing, power penetration, gas turbines rotors, medical apparatus, etc. are the real-world applications of rotating fluids flow documented by researchers. The viscous fluid flow by rotating disk was initially reported by Karman [14]. The MHD slip flow with entropy generation analysis by rotating disk was deliberated by Rashidi et al. [15]. Sheikholeslami et al. numerically analyzed the nanofluid flow through rotating disk [16]. Xun et al. [17] scrutinized the heat transfer in a fluid flow due to rotating disk. Latiff et al. examined the bioconvective flow of fluid due to rotating disk [18]. Imtiaz et al. [19] determined the MHD slip flow by rotating disk. Doh and Muthtamilselvan [20] probed the MHD fluid flow by rotating disk. Ellahi et al. [21] deliberated the multi-fluid flow with nano-sized gold and silver particles by rotating disk. Hayat et al. [22] explored the MHD fluid flow with slip conditions by rotating disk. Bhatti et al. [23] analyzed the MHD non-Newtonian nanofluid with entropy generation over a shrinking surface. Shah et al. [24] deliberated the MHD thin film flow of nanofluid through a rotating disk. Dawar et al. [25] premeditated the flow of unsteady squeezing nanofluid in rotating channels. Dawar et al. [26] scrutinized the MHD thin film flow by a rotating disk. Recently, Asma et al. analyzed the flow of nanofluid with chemical reaction [27]. Others related articles can be seen in [28,29,30,31,32].

The procedure of heat transmission in engineering and industrial processes is exceedingly dependent on the structure of the surface from which heat transfer occurs to the fluid. The phenomenon of heat transmission occurs due to temperature differences. The heat transfer process can be studied via convective boundary condition, constant or prescribed surface temperature, constant or prescribed heat flux, and Newtonian heating. Vo et al. studied heat transport in the flow of nanomaterial with porous medium over a permeable stretched sheet [33]. Sheikholeslami et al. [34,35] examined magnetohydrodynamic flow of heated nanofluid with thermal radiation in a porous enclosure. They used numerical approached. Recent study about heat transfer and nanofluid with different approached in different geometries can be seen [36,37,38,39].

Here, in this article, we have presented the MHD nanofluid flow through a porous rotating disk with slip conditions. The impact of heat source sink is also studied. The nanofluid flow is analyzed with thermophoresis and Brownian motion impacts. The joule dissipation influence is also taken in this nanofluid flow phenomenon. Analytical and numerical approaches are applied to examine the modeled problem and also compared each other, and good results were obtained.

## 2. Problem Formulation

**Figure 1:**
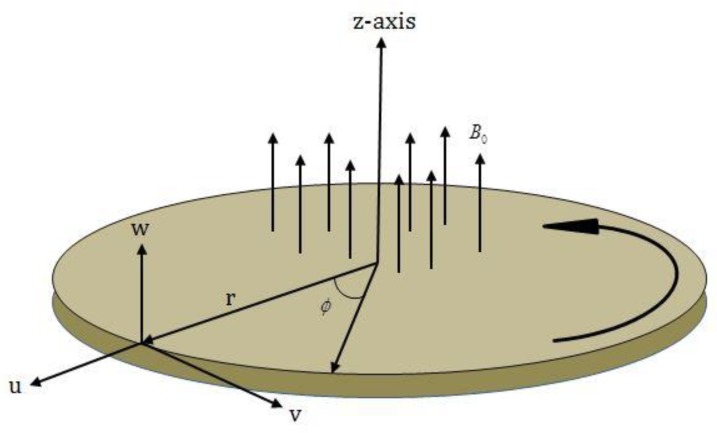
Fluid flow geometry.

The MHD nanofluid flow subject to velocity slip conditions is considered here. The nanofluid flow is considered as time dependent and incompressible. The flow is studied over a rotating porous disk. The disk rotates along z-axis with angular velocity Ω (see Figure 1). The magnetic field is functional along the z-direction. The electric and Hall current influences are ignored throughout the study. The fluid flow is treated with viscous dissipation impact. The heat and mass transmission characteristics are analyzed in the presence of thermophoresis and Brownian motion impacts. The nanofluid flow is based on the present situations [5,22,29]:

(1) $$u_{r}+\frac{u}{r}+w_{z}=0,$$

(2) $$uu_{r}-\frac{v^{2}}{r}+wu_{z}=\upsilon \left(u_{rr}+\frac{u_{r}}{r}-\frac{u}{r^{2}}+u_{zz}\right)-\left(\frac{\sigma B_{0}^{2}}{\rho_{f}}u+\frac{\upsilon }{K}u\right),$$

(3) $$uv_{r}+\frac{uv}{r}+wv_{z}=\upsilon \left(v_{rr}+\frac{v_{r}}{r}-\frac{v}{r^{2}}+v_{zz}\right)-\left(\frac{\sigma B_{0}^{2}}{\rho_{f}}v+\frac{\upsilon }{K}v\right),$$

(4) $$uw_{r}+ww_{z}=\upsilon \left(w_{rr}+\frac{w_{r}}{r}+w_{zz}\right),$$

(5) $$\begin{array}{l} uT_{r}+wT_{z}=\alpha \left(T_{rr}+\frac{T_{r}}{r}+T_{zz}\right)+Q_{0}\left(T-T_{\infty }\right)+\frac{\sigma B_{0}^{2}}{\rho_{f}}\left(u^{2}+v^{2}\right)\\ +\frac{{\left(\rho c\right)}_{p}}{{\left(\rho c\right)}_{f}}\left[D_{B}\left(T_{z}C_{z}+T_{r}C_{r}\right)\right]+\frac{{\left(\rho c\right)}_{p}}{{\left(\rho c\right)}_{f}}\left[\frac{D_{T}}{T_{\infty }}\left\{{\left(T_{z}\right)}^{2}+{\left(T_{r}\right)}^{2}\right\}\right], \end{array}$$

(6) $$uC_{r}+wC_{z}=D_{B}\left(C_{zz}+\frac{C_{r}}{r}+C_{rr}\right)+\frac{D_{T}}{T_{\infty }}\left(T_{zz}+\frac{T_{r}}{r}+T_{rr}\right),$$

The consistent boundary conditions are

(7) $$\begin{array}{l} u=Lu_{z},\;v=\Omega r+Lv_{z},\;w=0,\;T=T_{w},\;C=C_{w}\;\mathrm{at}\;z=0,\\ u\to 0,\;v\to 0,\;T\to T_{\infty },\;C\to C_{\infty }\;\mathrm{as}\;z\to \infty . \end{array}$$

The similarity transformations are defined as

(8) $$u=\Omega rf^{\prime }\left(\xi \right),\;v=\Omega rg\left(\xi \right),\;w=-{\left(2\Omega \upsilon \right)}^{\frac{1}{2}}f\left(\xi \right),\theta \left(\xi \right)=\frac{T-T_{\infty }}{T_{w}-T_{\infty }},\;\phi \left(\xi \right)=\frac{C-C_{\infty }}{C_{w}-C_{\infty }},\;\xi ={\left(\frac{2\Omega }{\upsilon }\right)}^{\frac{1}{2}}z.$$

Using (8), (1) satisfies, and ((2)–(7)) are reduced as

(9) $$2f^{\prime \prime \prime }+2ff^{\prime \prime }+g^{2}-{\left(f^{\prime }\right)}^{2}-Mf^{\prime }-\kappa f^{\prime }=0,$$

(10) $$2g^{\prime \prime }+2fg^{\prime }-2gf^{\prime }-Mg-\kappa g=0,$$

(11) $$\frac{1}{\mathrm{Pr}}\theta^{\prime \prime }+f\theta^{\prime }+Nb\theta^{\prime }\phi^{\prime }+Nt{\left(\theta^{\prime }\right)}^{2}+\gamma \theta +MEc\left\{{\left(f^{\prime }\right)}^{2}+{\left(g\right)}^{2}\right\}=0,$$

(12) $$\phi^{\prime \prime }+Le\mathrm{Pr}f\phi^{\prime }+\frac{Nt}{Nb}\theta^{\prime \prime }=0,$$

(13) $$\begin{array}{l} f=0,\;f^{\prime }=\psi f^{\prime \prime },\;g=1+\psi g^{\prime },\;\theta =1,\;\phi =1\;\mathrm{at}\;\xi =0,\\ f^{\prime }\to 0,\;g\to 0,\;\theta \to 0,\;\phi \to 0\;\mathrm{as}\;\xi \to \infty . \end{array}$$

where the dimensionless parameters are defined as:

(14) $$\begin{array}{l} M=\sqrt{\frac{\sigma B_{0}^{2}}{\rho_{f}\Omega }},\;\kappa =\frac{\upsilon }{k\Omega },\;\mathrm{Pr}=\frac{\upsilon }{\alpha },\;\gamma =\frac{Q_{0}}{2\Omega },\;Nb=\frac{{\left(\rho c\right)}_{p}}{{\left(\rho c\right)}_{f}}\frac{\left(T_{w}-T_{\infty }\right)D_{T}}{T_{\infty }\upsilon },\;\\ Le=\frac{\alpha }{D_{B}},\;\psi =L{\left(\frac{2\Omega }{\upsilon }\right)}^{\frac{1}{2}},Nt=\frac{{\left(\rho c\right)}_{p}}{{\left(\rho c\right)}_{f}}\frac{\left(C_{w}-C_{\infty }\right)D_{B}}{\upsilon },\;Ec=\frac{{\left(r\Omega \right)}^{2}}{\left(T_{w}-T_{\infty }\right)}\;. \end{array}$$

The dimensionless surface quantities are defined as

(15) RerCf=f″(0),    RerCg=   g′(0),   1RerNu=−θ′(0),     1RerSh=−ϕ′(0), 

Entirely the overhead factors are defined in nomenclature.

## 3. Analytical Solution

Here, the proposed model is elucidated by using HAM [40,41,42,43]. In view of ((9)–(12)) with (13); the primary assumptions are deliberated as:

(16) $$f_{0}(\xi )=0,\;g_{0}(\xi )=\frac{1}{1+\psi }e^{-\xi },\;\theta_{0}(\xi )=e^{-\xi },\;\phi_{0}(\xi )=e^{-\xi }.$$

The $L_{f}{,L}_{g}{,L}_{\theta }$ and ${\;L}_{\phi }$ are picked as:

(17) $$L_{f}\left(f\right)=f^{\prime \prime \prime }-f^{\prime },\;L_{g}\left(g\right)=g^{\prime \prime }-g,\;L_{\theta }\left(F\right)=\theta^{\prime \prime }-\theta ,\;L_{\phi }\left(\phi \right)=\phi^{\prime \prime }-\phi ,$$

with the following properties:

(18) $$L_{f}\left(m_{1}+m_{2}e^{-\xi }+m_{3}e^{\xi }\right)=0,{\;L}_{g}\left(m_{4}e^{-\xi }+m_{5}e^{\xi }\right)=0,L_{\theta }\left(m_{6}e^{-\xi }+m_{7}e^{\xi }\right)=0,{\;L}_{\phi }\left(m_{8}e^{-\xi }+m_{9}e^{\xi }\right)=0,$$

where $m_{i}(i=1-9)$ are constants.

The resultant non-linear operators $N_{f},\;N_{g},\;N_{\theta }$, and $N_{\phi }$ are indicated as:

(19) $$\begin{array}{l} N_{f}\left[f(\xi ;\tau ),\;g(\xi ;\tau )\right]=2\frac{\partial^{3}f(\xi ;\tau )}{\partial \xi^{3}}+2f(\xi ;\tau )\frac{\partial^{2}f(\xi ;\tau )}{\partial^{2}\xi }\\ +{\left(g\left(\xi ;\tau \right)\right)}^{2}-{\left(\frac{\partial f(\xi ;\tau )}{\partial \xi }\right)}^{2}-M\frac{\partial f(\xi ;\tau )}{\partial \xi }-\kappa \frac{\partial f(\xi ;\tau )}{\partial \xi }, \end{array}$$

(20) $$N_{g}\left[g(\xi ;\tau ),\;f(\xi ;\tau )\right]=2\frac{\partial^{2}g(\xi ;\tau )}{\partial \xi^{2}}+2f(\xi ;\tau )\frac{\partial g(\xi ;\tau )}{\partial \xi }-2g(\xi ;\tau )\frac{\partial f(\xi ;\tau )}{\partial \xi }-Mg(\xi ;\tau )-\kappa g(\xi ;\tau ),$$

(21) $$\begin{array}{l} N_{\theta }\left[\theta (\xi ;\tau ),\;f(\xi ;\tau ),\;g(\xi ;\tau ),\;\phi (\xi ;\tau )\right]=\frac{1}{\mathrm{Pr}}\frac{\partial^{2}\theta (\xi ;\tau )}{\partial \xi^{2}}+f(\xi ;\tau )\frac{\partial \theta (\xi ;\tau )}{\partial \xi }+\\ Nb\frac{\partial \theta (\xi ;\tau )}{\partial \xi }\frac{\partial \phi (\xi ;\tau )}{\partial \xi }+Nt{\left(\frac{\partial \theta (\xi ;\tau )}{\partial \xi }\right)}^{2}+\gamma \theta (\xi ;\tau )+MEc\left\{{\left(\frac{\partial f(\xi ;\tau )}{\partial \xi }\right)}^{2}+{\left(g(\xi ;\tau )\right)}^{2}\right\}, \end{array}$$

(22) $$N_{\phi }\left[\phi (\xi ;\tau ),\;f(\xi ;\tau ),\;\theta (\xi ;\tau )\right]=\frac{\partial^{2}\phi (\xi ;\tau )}{\partial \xi^{2}}+Le\mathrm{Pr}+f(\xi ;\tau )\frac{\partial \phi (\xi ;\tau )}{\partial \xi }+\frac{Nt}{Nb}\frac{\partial^{2}\theta (\xi ;\tau )}{\partial \xi^{2}}.$$

The zeroth-order problem is

(23) $$(1-\tau )L_{f}\left[f(\xi ;\tau )-f_{0}(\xi )\right]=\tau \hbar_{f}N_{f}\left[f(\xi ;\tau ),\;g(\xi ;\tau )\right],$$

(24) $$(1-\tau )L_{g}\left[g(\xi ;\tau )-g_{0}(\xi )\right]=\tau \hbar_{g}N_{g}\left[g(\xi ;\tau ),\;g(\xi ;\tau )\right],$$

(25) $$(1-\tau )L_{\theta }\left[\theta (\xi ;\tau )-\theta_{0}(\xi )\right]=\tau \hbar_{\theta }N_{\theta }\left[\theta (\xi ;\tau ),\;f(\xi ;\tau ),\;g(\xi ;\tau ),\;\phi (\xi ;\tau )\right],$$

(26) $$(1-\tau )L_{\phi }\left[\phi (\xi ;\tau )-\phi_{0}(\xi )\right]=\tau \hbar_{\phi }N_{\phi }\left[\phi (\xi ;\tau ),\;f(\xi ;\tau ),\;g(\xi ;\tau ),\;\theta (\xi ;\tau )\right].$$

The equivalent boundary conditions are:

(27) $$\begin{array}{l} {f(\xi ;\tau )|}_{\xi =0}=0,\;{\frac{\partial f(\xi ;\tau )}{\partial \xi }|}_{\xi =0}=\psi \frac{\partial^{2}f(\xi ;\tau )}{\partial \xi^{2}},\;{\frac{\partial f(\xi ;\tau )}{\partial \xi }|}_{\xi \to \infty }=0,\\ \;{g(\xi ;\tau )|}_{\xi =0}=1+\psi \frac{\partial g(\xi ;\tau )}{\partial \xi },\;{g(\xi ;\tau )|}_{\xi \to \infty }=0,\\ {\theta (\xi ;\tau )|}_{\xi =0}=1,\;{\theta (\xi ;\tau )|}_{\xi \to \infty }=0,\;\\ {\phi (\xi ;\tau )|}_{\xi =0}=1,\;{\phi (\xi ;\tau )|}_{\xi \to \infty }=0, \end{array}$$

where τ∈[0,1] is the imbedding parameter and $\hbar_{f},\;\hbar_{g},\;\hbar_{\theta }$, and $\hbar_{\phi }$ are used to regulate the convergence of the solution. When τ=0 and τ=1, we have:

(28) $$\begin{array}{l} f(\xi ;0)=f_{0}(\xi ),\;f(\xi ;1)=f(\xi ),\;\\ g(\xi ;0)=g_{0}(\xi ),\;g(\xi ;1)=g(\xi ),\\ \theta (\xi ;0)=\theta_{0}(\xi ),\;\theta (\xi ;1)=\theta (\xi ),\;\\ \phi (\xi ;0)=\phi_{0}(\xi ),\;\phi (\xi ;1)=\phi (\xi ), \end{array}$$

Expanding f(ξ;τ),g(ξ;τ),θ(ξ;τ) and ϕ(ξ;τ) by Taylor’s series

(29) $$\begin{array}{l} f(\xi ;\tau )=f_{0}(\xi )+\sum_{q=1}^{\infty }f_{q}(\xi )\tau^{q},\;\\ g(\xi ;\tau )=g_{0}(\xi )+\sum_{q=1}^{\infty }g_{q}(\xi )\tau^{q},\\ \theta (\xi ;\tau )=\theta_{0}(\xi )+\sum_{q=1}^{\infty }\theta_{q}(\xi )\tau^{q},\\ \phi (\xi ;\tau )=\;\phi_{0}(\xi )+\sum_{q=1}^{\infty }\phi_{q}(\xi )\tau^{q}. \end{array}$$

where

(30) $$\begin{array}{l} f_{q}(\xi )={\frac{1}{q!}\frac{\partial f(\xi ;\tau )}{\partial \xi }|}_{\tau =0},g_{q}(\xi )={\frac{1}{q!}\frac{\partial g(\xi ;\tau )}{\partial \xi }|}_{\tau =0},\;\theta_{q}(\xi )={\frac{1}{q!}\frac{\partial \theta (\xi ;\tau )}{\partial \xi }|}_{\tau =0}\;\\ \mathrm{and}\;\;\phi_{q}(\xi )={\frac{1}{q!}\frac{\partial \phi (\xi ;\tau )}{\partial \xi }|}_{\tau =0}. \end{array}$$

The secondary constraints $\hbar_{f},\hbar_{g},\;\hbar_{\theta }$, and $\hbar_{\phi }$ are selected, such that the series (29) converges at τ=1, changing τ=1 in (29), we get:

(31) $$\begin{array}{l} f(\xi )=f_{0}(\xi )+\sum_{q=1}^{\infty }f_{q}(\xi ),\;\\ g(\xi )=g_{0}(\xi )+\sum_{q=1}^{\infty }g_{q}(\xi ),\\ \theta (\xi )=\theta_{0}(\xi )+\sum_{q=1}^{\infty }\theta_{q}(\xi ),\;\\ \phi (\xi )=\;\phi_{0}(\xi )+\sum_{q=1}^{\infty }\phi_{q}(\xi ). \end{array}$$

The $q^{th}$-order problem satisfies the following:

(32) $$\begin{array}{l} L_{f}\left[f_{q}(\xi )-\chi_{q}f_{q-1}(\xi )\right]=\hbar_{f}U_{q}^{f}(\xi ),\\ L_{g}\left[g_{q}(\xi )-\chi_{q}g_{q-1}(\xi )\right]=\hbar_{g}U_{q}^{g}(\xi ),\\ L_{\theta }\left[\theta_{q}(\xi )-\chi_{q}\theta_{q-1}(\xi )\right]=\hbar_{\theta }U_{q}^{\theta }(\xi ),\\ L_{\phi }\left[\phi_{q}(\xi )-\chi_{q}\phi_{q-1}(\xi )\right]=\hbar_{\phi }U_{q}^{\phi }(\xi ). \end{array}$$

The equivalent boundary conditions are:

(33) $$\begin{array}{l} f_{q}(0)={f^{\prime }}_{q}(0)-\psi {f^{\prime \prime }}_{q}(0)={f^{\prime }}_{q}(\infty )=0,\\ g_{q}(0)-\psi {g^{\prime }}_{q}(0)-1=g_{q}(\infty )=0,\\ \theta_{q}(0)=\theta_{q}(\infty )=0,\\ \theta_{q}(0)=\theta_{q}(\infty )=0. \end{array}$$

Here

(34) $$U_{q}^{f}(\xi )=2{f^{\prime \prime \prime }}_{q-1}+2\sum_{k=0}^{q-1}f_{q-1-k}{f^{\prime \prime }}_{k}+{\left(g_{q-1}\right)}^{2}-{\left({f^{\prime }}_{q-1}\right)}^{2}-M{\left({f^{\prime }}_{q-1}\right)}^{2}-\kappa {\left({f^{\prime }}_{q-1}\right)}^{2},$$

(35) $$U_{q}^{g}(\xi )=2{g^{\prime \prime }}_{q-1}+2f_{q-1}{g^{\prime }}_{q-1}-2g_{q-1}{f^{\prime }}_{q-1}-Mg_{q-1}-\kappa g_{q-1},$$

(36) $$U_{q}^{\theta }(\xi )=\frac{1}{\mathrm{Pr}}{\theta^{\prime \prime }}_{q-1}+\sum_{k=0}^{q-1}f_{q-1-k}{\theta^{\prime }}_{k}+Nb\sum_{k=0}^{q-1}{\theta^{\prime }}_{q-1-k}{\phi^{\prime }}_{k}+Nt{\left({\theta^{\prime }}_{q-1}\right)}^{2}+\gamma \theta_{q-1}+MEc\left\{{\left({f^{\prime }}_{q-1}\right)}^{2}+{\left(g_{q-1}\right)}^{2}\right\},$$

(37) $$U_{q}^{\phi }(\xi )={\phi^{\prime \prime }}_{q-1}+Le\mathrm{Pr}\sum_{k=0}^{q-1}f_{q-1-k}{\phi^{\prime }}_{k}+\frac{Nt}{Nb}{\theta^{\prime \prime }}_{q-1},$$

where

(38) $$\chi_{q}=\left\{ \begin{array}{l} 0,\;\mathrm{if}\;\tau \leq 1\\ 1,\;\mathrm{if}\;\tau >1 \end{array}\right.$$

## 4. Convergence Solution

**Figure 2:**
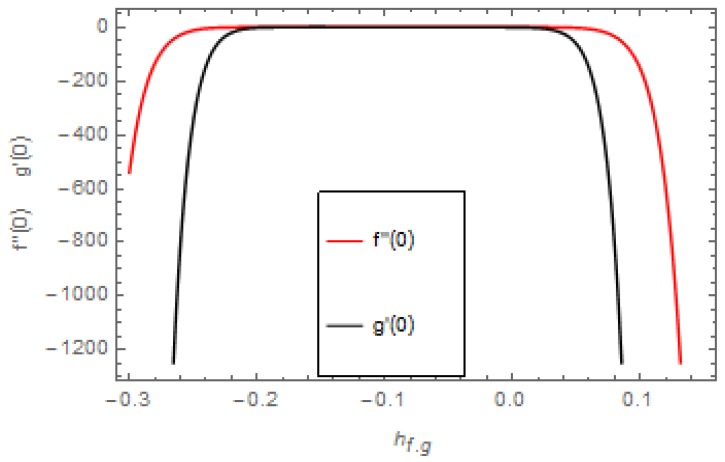
Curves for velocities profiles $f^{\prime \prime }\left(0\right)$ and $g^{\prime }\left(0\right)$.

**Figure 3:**
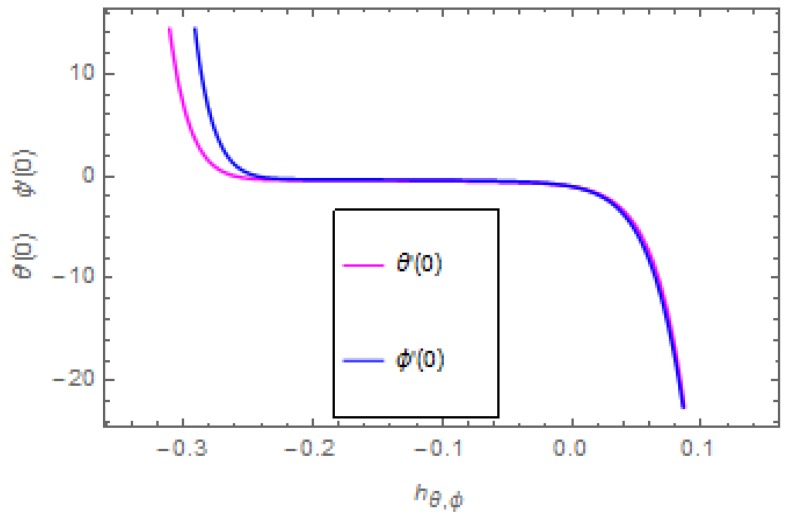
Curves for temperature and concentration profiles $\phi^{\prime }\left(0\right)$ and $\theta^{\prime }\left(0\right)$.

HAM guarantees the convergence of the series solution of the modeled problem. The auxiliary parameter ħ plays an important role in adjusting the region of convergence of the series solution. Figure 2 indicates the ħ-curves of the velocities profiles. The auxiliary parameters $\hbar_{f}$ and $\hbar_{g}$ are $-0.26\leq \hbar_{f}\leq 0.1$ and $-0.22\leq \hbar_{g}\leq 0.06$. Figure 3 indicates the ħ-curves of the temperature and concentration profiles. The auxiliary parameters $\hbar_{\theta }$ and $\hbar_{\phi }$ are $-0.28\leq \hbar_{f}\leq 0.02$ and $-0.24\leq \hbar_{g}\leq 0.02$.

## 5. Results and Discussion

**Figure 4:**
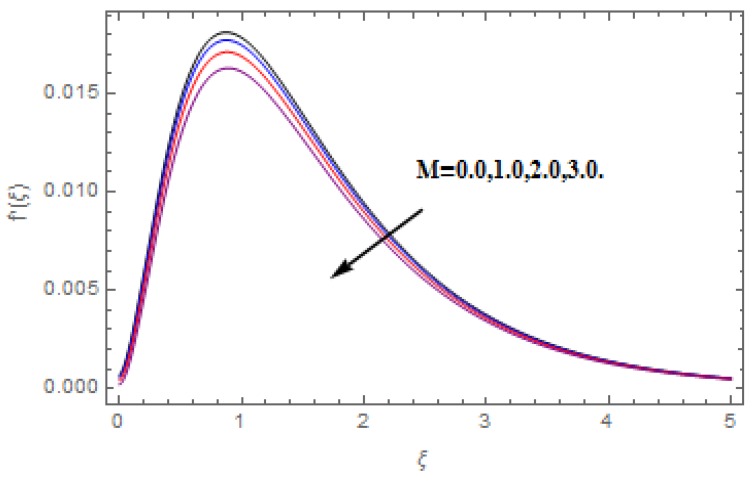
Varation in $f^{\prime }\left(\xi \right)$ against M, when ψ=0.2, Nt=Nb=0.5,  Le=1.0,   Ec=1.0,  Pr=1.2,   γ=0.1,   κ=1.0.

**Figure 5:**
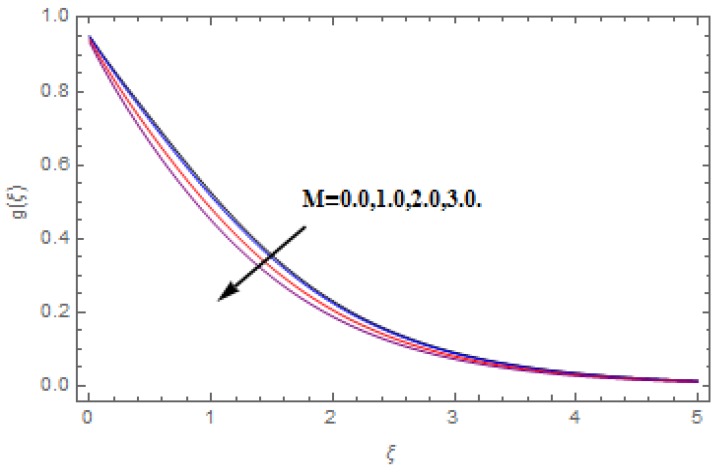
Varation in g(ξ) against M, when ψ=0.2, Nt=Nb=0.5,  Le=1.0,   Ec=1.0,  Pr=1.2,   γ=0.1,   κ=1.0.

**Figure 6:**
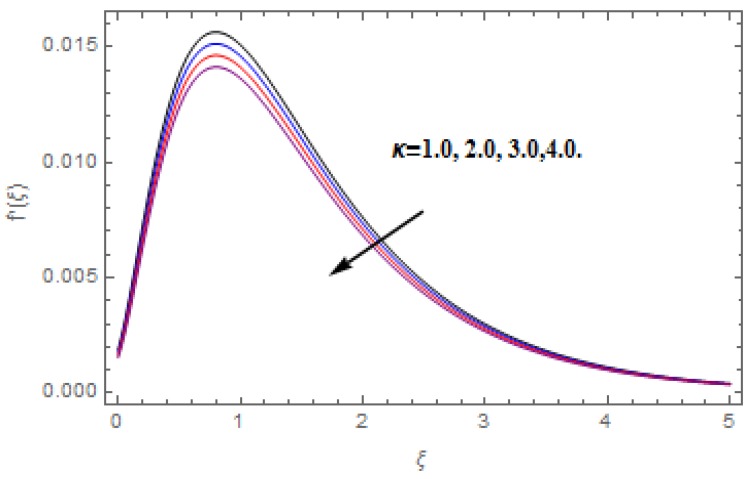
Varation in $f^{\prime }\left(\xi \right)$ gainst κ, when M=ψ=0.2, Nt=Nb=0.5,  Le=1.0,   Ec=1.0,  Pr=1.2,   γ=0.1.

**Figure 7:**
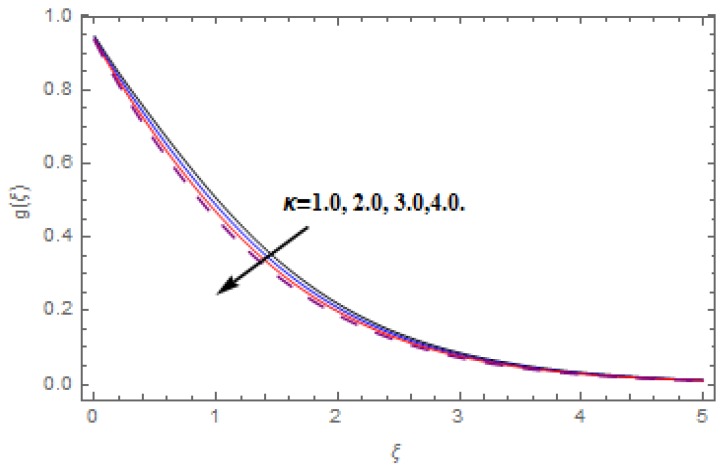
Varation in g(ξ) against κ, when M=ψ=0.2, Nt=Nb=0.5,  Le=1.0,   Ec=1.0,  Pr=1.2,   γ=0.1.

**Figure 8:**
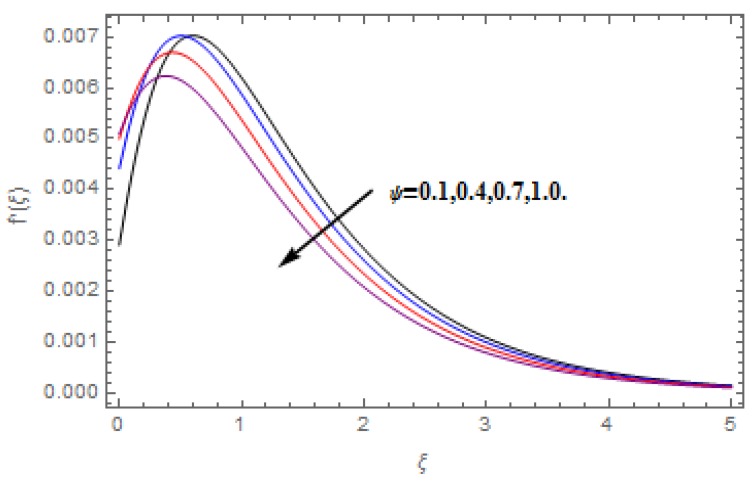
Varation in $f^{\prime }\left(\xi \right)$ against ψ, when M=0.2, Nt=Nb=0.5,  Le=1.0,   Ec=1.0,  Pr=1.2,   γ=0.1,   κ=1.0.

**Figure 9:**
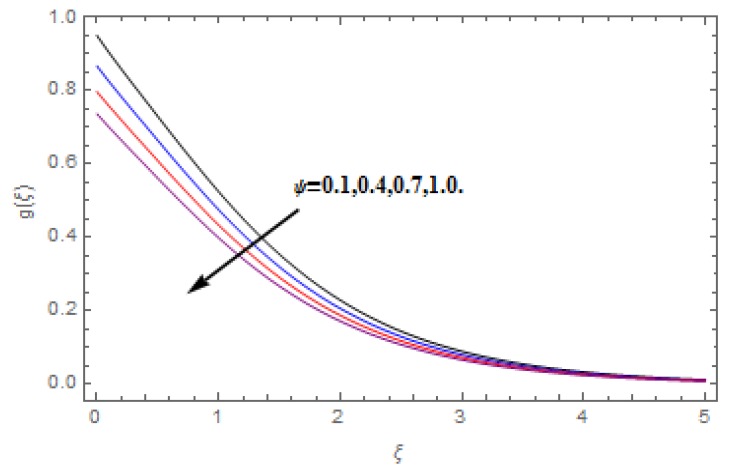
Varation in g(ξ) against ψ, when M=0.2, Nt=Nb=0.5,  Le=1.0,   Ec=1.0,  Pr=1.2,   γ=0.1,   κ=1.0.

**Figure 10:**
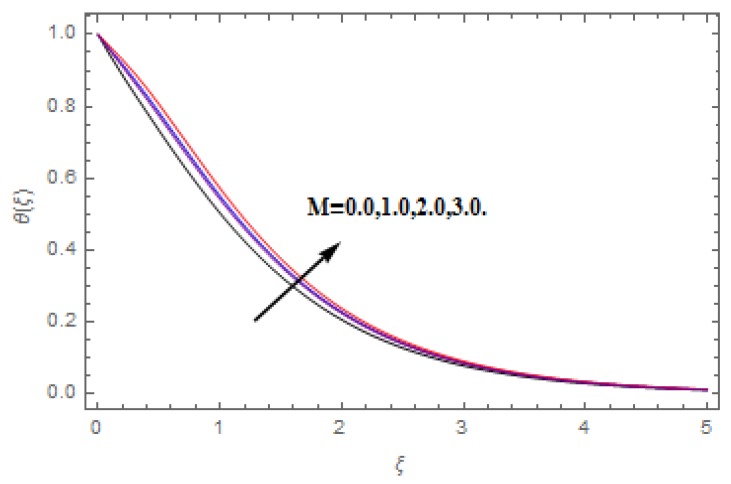
Varation in θ(ξ) against M, when ψ=0.2, Nt=Nb=0.5,  Le=1.0,   Ec=1.0,  Pr=1.2,   γ=0.1,   κ=1.0.

**Figure 11:**
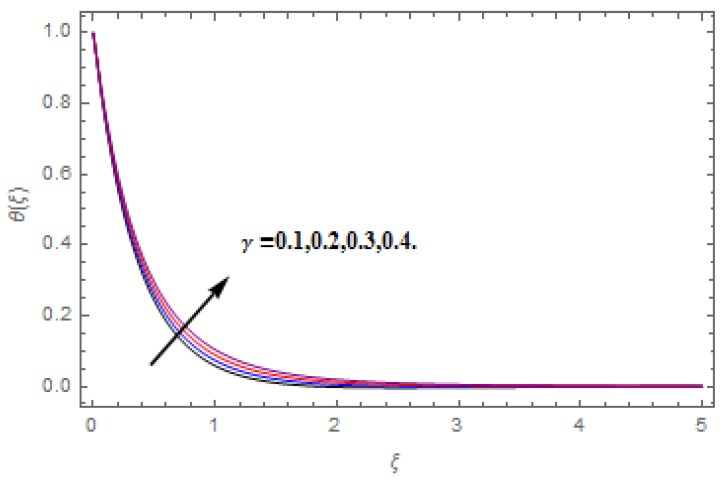
Varation in θ(ξ) against γ, when M=ψ=0.2, Nt=Nb=0.5,  Le=1.0,   Ec=1.0,  Pr=1.2,   κ=1.0.

**Figure 12:**
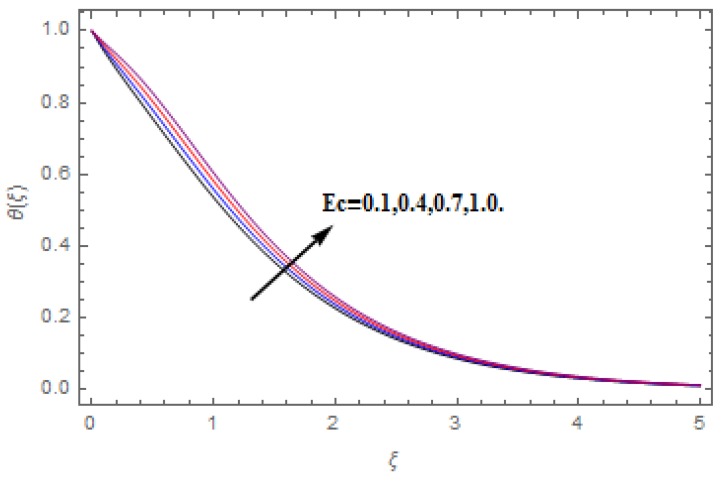
Varation in θ(ξ) against Ec, when M=ψ=0.2, Nt=Nb=0.5,  Le=1.0,  Pr=1.2,   γ=0.1,   κ=1.0.

**Figure 13:**
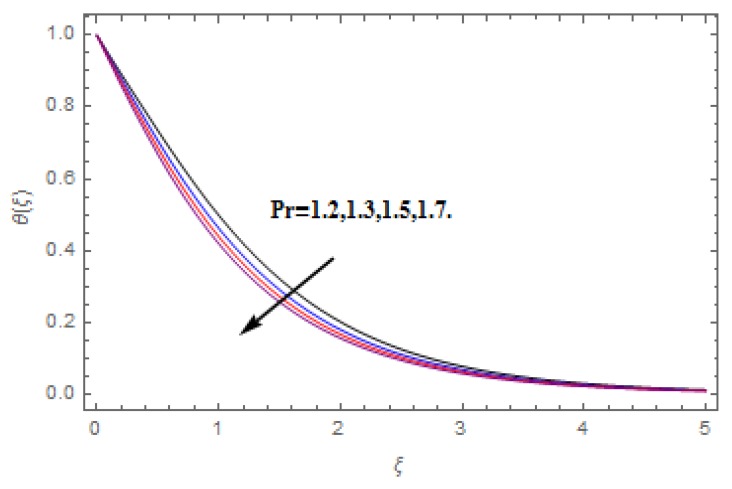
Varation in θ(ξ) against Pr, when M=ψ=0.2, Nt=Nb=0.5,  Le=1.0,   Ec=1.0,   γ=0.1,   κ=1.0.

**Figure 14:**
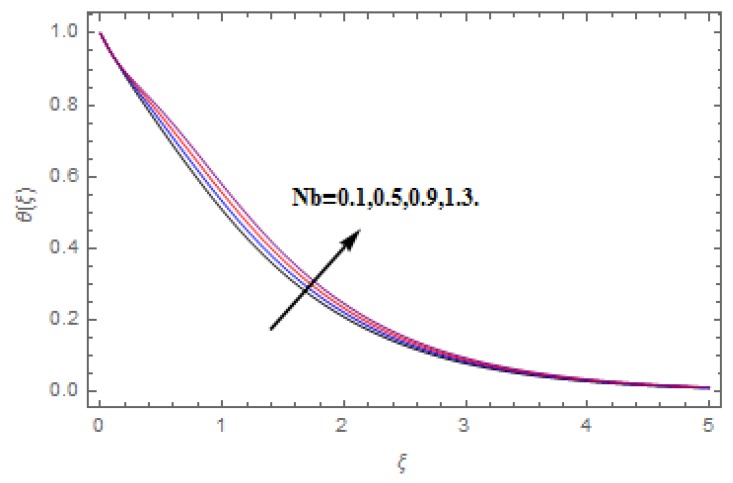
Varation in θ(ξ) against Nb, when M=ψ=0.2, Nt=0.5,  Le=1.0,   Ec=1.0,  Pr=1.2,   γ=0.1,   κ=1.0.

**Figure 15:**
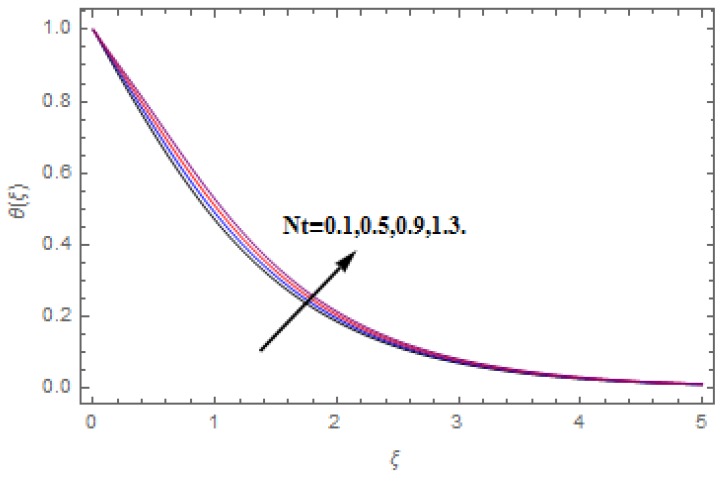
Varation in θ(ξ) against Nt, when M=ψ=0.2,  Nb=0.5,  Le=1.0,   Ec=1.0,  Pr=1.2,   γ=0.1,   κ=1.0.

**Figure 16:**
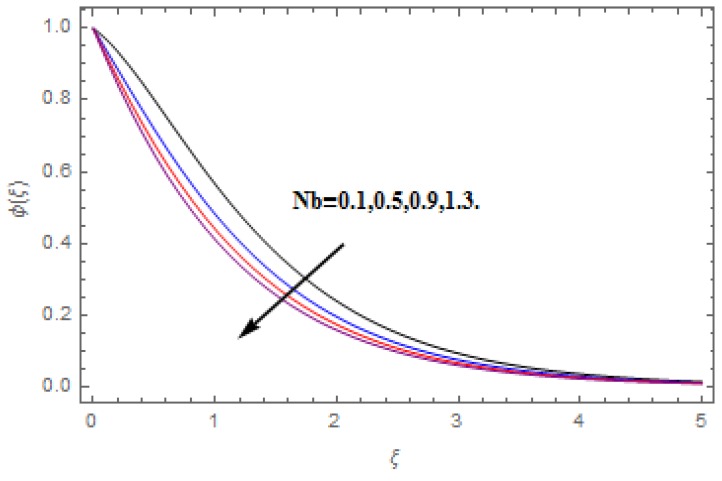
Varation in ϕ(ξ) against Nb, when M=ψ=0.2, Nt=0.5,  Le=1.0,   Ec=1.0,  Pr=1.2,   γ=0.1,   κ=1.0.

**Figure 17:**
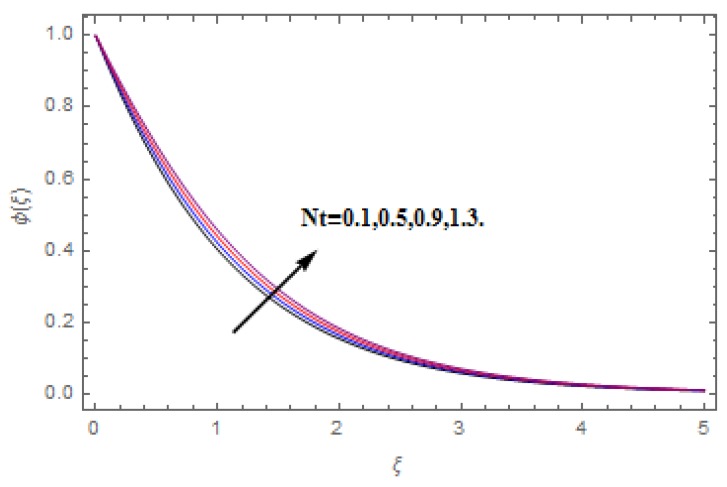
Varation in ϕ(ξ) against Nt, when M=ψ=0.2,  Nb=0.5,  Le=1.0,   Ec=1.0,  Pr=1.2,   γ=0.1,   κ=1.0.

**Figure 18:**
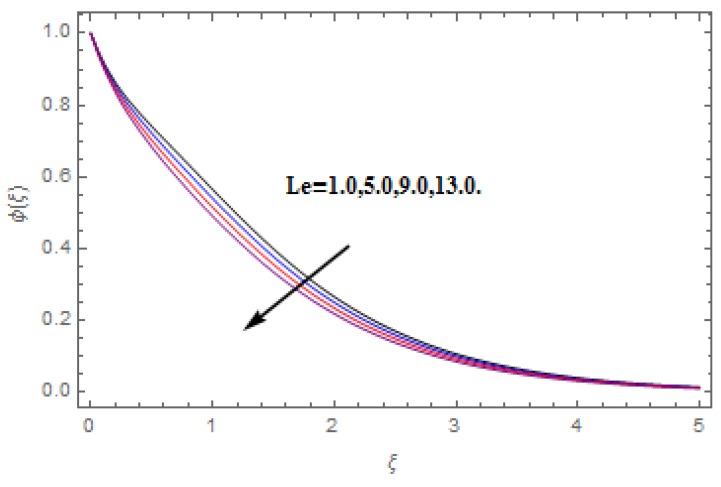
Varation in ϕ(ξ) against Le, when M=ψ=0.2, Nt=Nb=0.5,  Ec=1.0,  Pr=1.2,   γ=0.1,   κ=1.0.

The aim of this section is to visualize variations in velocities, temperature, concentration, Nusselt number, and skin friction coefficient due to involved parameters, like magnetic field (M), porosity (κ), velocity slip (ψ), Eckert number (Ec), heat source/sink (γ), thermophoresis (Nt), Prandtl number (Pr), Lewis number (Le), and Brownian motion (Nb) developed during the nanofluid flow that are displayed in Figure 4, Figure 5, Figure 6, Figure 7, Figure 8, Figure 9, Figure 10, Figure 11, Figure 12, Figure 13, Figure 14, Figure 15, Figure 16, Figure 17 and Figure 18. Figure 4 and Figure 5 depict the reducing influence of M on $f^{\prime }(\xi )$ and g(ξ). The increasing M causes deterioration in momentum boundary layer thickness and velocity profiles. M relates with the Lorentz force theory. The Lorentz force always creates conflicting force to the flow of fluid and decays motion of the fluid particles. Accordingly, the escalating magnetic force declines the fluid velocity. The escalating κ declines $f^{\prime }(\xi )$ and g(ξ) is depicted in Figure 6 and Figure 7. The porous media usually performs opposite behavior to the fluid flow. With an increase in the porous media, the fluid particles motion reduces and, thus, the fluid velocity diminishes. Therefore, the growing estimations of κ diminishes $f^{\prime }(\xi )$ and g(ξ). Figure 8 and Figure 9 depict the escalating ψ diminishes $f^{\prime }(\xi )$ and g(ξ). The velocity slip parameter always performs a reverse impact on velocity profiles. The corresponding boundary layer thickness declines by ψ, which deescalates $f^{\prime }(\xi )$ and g(ξ). Figure 10 depicts the impression of M on θ(ξ). It is witnessed that the escalating M escalates θ(ξ). The influence of γ on θ(ξ) is demonstrated in Figure 11. The heat source/sink plays like heat producer. As the parameter estimations intensify, the fluid particles temperature heightens. For that reason θ(ξ) upsurges. Figure 12 portrays the effect of Ec on θ(ξ). It is used for extremely fast compressible flow. The positive Eckert number represents the freezing of wall and, as a result, the convection of heat transmission to the fluid is augmented. Figure 13 shows the consequence of Pr on θ(ξ). Pr makes the association of fluid viscosity with thermal conductivity. The fluids have high thermal conductivity with large Pr, while the impact is reverse for higher Pr. Hence, the escalating estimations of Pr deescalates θ(ξ). Figure 14 illustrates the effect of Nb on θ(ξ). Higher Brownian motion induces the random acceleration of the fluid particles. Extra energy is generated because of this random acceleration. Therefore, the thermal rise is reported. Figure 15 presents the impression of Nt on θ(ξ). In the thermophoresis phenomenon, tiny fluid particles are forced back from those in the warmer to the cold surface. As a result, the fluid particles returned from those in the warmed surface and the thermal curve then increased. The outcome of Nb and Nt on ϕ(ξ) are shown in Figure 16 and Figure 17. The higher estimations of Nb shows reverse impact on ϕ(ξ). Figure 17 illustrates the rising impression of Nt on ϕ(ξ). Figure 18 demonstrates the influence of Le on ϕ(ξ). Le is the correlation of mass diffusion to fluid thermal conductivity. The increasing Le causes thickness of the concentration layer, which consequently escalates the concentration profile.

Table 1, Table 2 and Table 3 are displayed to examine the surface drag force, heat transfer rate, and mass transfer rate, respectively. Table 1 depicts that the increasing M and κ reduce both $C_{f}$ and $C_{g}$, while the rising values of ψ reduces $C_{f}$ and increases $C_{g}$. From Table 2, the escalating M, ψ, Le, Nb and Nt reduces Nu, while the higher Pr escalates the Nu. From Table 3, M and ψ deescalate Sh, while the higher Le, Pr, Nb, and Nt escalate Sh. Table 4 and Table 5 show the comparison of HAM and Shooting approaches for velocities, temperature, and concentration profiles. Both of the techniques are treated with the established computer codes and validated by publishing the results to the accessible standard and they have computed with software mathematica. At 20th order of approximations, the results of HAM have been computed. Here, the validity of the proposed model is observed.

## 6. Conclusions

The steady and incompressible flow of MHD nanofluid over a porous rotating disc with slip conditions is examined. The mass and heat transmission with viscous dissipation impact is also intentional. The problem is solved with the help of analytical and numerical methods. The core points of the current inspection are mentioned beneath:

❖ Increasing magnetic, velocity slip, and porosity parameters perform reducing behavior on velocities profiles.
❖ Increasing Eckert number, thermophoresis, Brownian motion, magnetic, and heat source/sink parameters perform reducing behavior on temperature profile while the Prandtl number performs opposite conduct on temperature profile.
❖ Increasing thermophoresis parameter performs increasing behavior on concentration profile, while the Brownian motion and Lewis number perform reducing behavior on concentration profile.
❖ The numerical and analytical approaches both agreed on the validation of the modeled problem.

## Figures and Tables

**Table 1:** Results of $C_{f}$ and $C_{g}$ against M, ψ, and κ.

M	ψ	κ	$C_{f}$	$C_{g}$
0.0	0.7	0.5	0.110921	−1.038800
0.7			0.096164	−1.135091
1.4			0.071695	−1.377442
0.3	0.2	0.5	0.204806	−1.265867
	0.5		0.136281	−1.133058
	0.8		0.094705	−1.022374
0.3	0.7	0.6	0.103762	−1.077193
		0.8	0.098619	−1.116139
		1.0	0.093783	−1.153702

**Table 2:** Results of Nu against M, ψ, Le, Pr, Nb and Nt.

M	ψ	Le	Pr	Nb	Nt	Nu
0.0	0.7	0.8	1.0	0.3	0.2	0.304942
0.7						0.244218
1.4						0.175662
0.3	0.2	0.8	1.0	0.3	0.2	0.326557
	0.5					0.303604
	0.8					0.287156
0.3	0.7	0.5	1.0	0.3	0.2	0.296330
		1.0				0.289543
		1.5				0.283952
0.3	0.7	0.8	0.5	0.3	0.2	0.249898
			1.0			0.292115
			1.5			0.322861
0.3	0.7	0.8	1.0	0.5	0.2	0.263410
				0.7		0.236772
				1.0		0.200563
0.3	0.7	0.8	1.0	0.3	0.5	0.259131
					0.7	0.238654
					1.0	0.210105

**Table 3:** Results of Sh against M, ψ, Le, Pr, Nb, and Nt.

M	ψ	Le	Pr	Nb	Nt	Sh
0.0	0.7	0.8	1.0	0.3	0.2	0.270000
0.7						0.253871
1.4						0.237227
0.3	0.2	0.8	1.0	0.3	0.2	0.275832
	0.5					0.269335
	0.8					0.264939
0.3	0.7	0.5	1.0	0.3	0.2	0.264933
		1.0				0.213734
		1.5				0.301323
0.3	0.7	0.8	0.5	0.3	0.2	0.386903
			1.0			0.229347
			1.5			0.266243
0.3	0.7	0.8	1.0	0.5	0.2	0.312629
				0.7		0.393384
				1.0		0.478752
0.3	0.7	0.8	1.0	0.3	0.5	0.329593
					0.7	0.222062
					1.0	0.225392
0.3	0.7	0.8	1.0	0.3	0.2	0.228550

**Table 4:** Comparison of HAM and Shooting techniques for $f^{\prime }\left(\xi \right)$ and g(ξ) while considering other parameters constant (ψ=0.3, κ=0.8, M=0.6 ).

ξ	$f^{\prime }\left(\xi \right)$		g(ξ)	
	HAM Solution	Shooting Solution	HAM Solution	Shooting Solution
0.0	−0.025173	−0.025358	−0.364493	−0.373437
0.5	−0.028523	−0.028712	−1.177880	−1.190384
1.0	0.016743	−0.016840	−0.938948	−0.947851
1.5	−0.009319	−0.009366	−0.623657	−0.629256
2.0	−0.005282	−0.005307	−0.391893	−0.395249
2.5	−0.003064	−0.003078	−0.241243	−0.243298
3.0	−0.001808	−0.001853	−0.147222	−0.148561
3.5	−0.001078	−0.001083	−0.089652	−0.090400
4.0	−0.000647	−0.000650	−0.054470	−0.054923
4.5	−0.000390	−0.000391	−0.033069	−0.033343
5.0	−0.000235	−0.000236	−0.020068	−0.020234

**Table 5:** Comparison of HAM and Shooting techniques for θ(ξ) and ϕ(ξ) considering other parameters constant (M=0.6, Ec=0.2, Nb=0.4, Nt=0.5, γ=0.6,  Pr=7.0, Le=1.0 ).

ξ	θ(ξ)		ϕ(ξ)	
	HAM Solution	Shooting Solution	HAM Solution	Shooting Solution
0.0	0.000000	1.000000	1.000000	1.000000
0.5	0.574478	0.574313	0.529982	0.529510
1.0	0.339813	0.339696	0.294610	0.294223
1.5	0.203589	0.203481	0.169102	0.168819
2.0	0.122669	0.122605	0.099093	0.098905
2.5	0.074132	0.074092	0.058838	0.058817
3.0	0.044869	0.044845	0.035224	0.035248
3.5	0.027181	0.027160	0.021194	0.021148
4.0	0.016474	0.016465	0.012793	0.012764
4.5	0.009988	0.009982	0.007736	0.007719
5.0	0.006056	0.006053	0.004643	0.004673

## Nomenclature

${Β}_{0}$	Magnetic field $\left[{\mathrm{NmA}}^{-1}\right]$
C	Concentration
$C_{w}$	Surface concentration
$C_{\infty }$	Concentration away from the surface
$D_{B}$	Brownian coefficient $\left[m^{2}s^{-1}\right]$
$D_{T}$	Thermophoretic coefficient $\left[m^{2}s^{-1}\right]$
Ec	Eckert number
k	Thermal conductivity $\left[Wm^{-1}K^{-1}\right]$
L	Velocity slip constant
Rer	Local Reynolds number
T	Temperature [K]
$T_{w}$	Surface temperature
$T_{\infty }$	Temperature away from the surface
u,v,w	Components of velocity $\left[{\mathrm{ms}}^{-1}\right]$
r,ϕ,z	Coordinates [m]
$Q_{0}$	Heat flux $\left[Wm^{-2}\right]$
γ	Heat source/sink parameter
υ	Kinematic viscosity $\left[m^{2}s^{-1}\right]$
$\rho_{f}$	Density $\left[{\mathrm{Kgm}}^{-3}\right]$
σ	Electrical conductivity $\left[{\mathrm{Sm}}^{-1}\right]$
${\left(\rho c\right)}_{f}$	Fluid heat capacity
${\left(\rho c\right)}_{p}$	nanoparticles heat capacity
**Parameters**	
Le	Lewis number
M	Magnetic
Nb	Brownian motion
Nt	Thermophoresis
Pr	Prandtl number
